# A critical appraisal of COVID-19 as a nosocomial infection: an African perspective

**DOI:** 10.11604/pamj.2020.36.310.25010

**Published:** 2020-08-20

**Authors:** Elijah Kolawole Oladipo, Olumuyiwa Elijah Ariyo, Francis Ifedayo Ibukun, Oluwadamilola Gideon Osasona, Ayodeji Akinwumi Akinbodewa, Chukwuyem Abejegah, Julius Kola Oloke

**Affiliations:** 1 Department of Microbiology, Laboratory of Molecular Biology, Bioinformatics and Immunology, Adeleke University, Ede, Osun State, Nigeria,; 2 Genomics Unit, Helix Biogen Consult, Ogbomoso, Oyo State, Nigeria,; 3 Department of Medicine, Infectious Diseases and Tropical Medicine Unit, Federal Teaching Hospital, Ido-Ekiti, Ekiti State, Nigeria,; 4 Salvato Laboratory, Division of Infectious Agents and Cancer, Institute of Human Virology, University of Maryland School of Medicine, Baltimore, United States,; 5 African Center of Excellence for Genomics of Infectious Diseases, Redeemers University, Ede, Osun State, Nigeria,; 6 Department of Biological Sciences, Redeemers University, Ede, Osun State, Nigeria,; 7 Department of Medicine, Kidney Care Center, University of Medical Sciences Teaching Hospital, Ondo State, Nigeria,; 8 Infection Control and Research Center, Community Health Department, Federal Medical Center, Owo, Ondo State, Nigeria,; 9 Department of Natural Science, Precious Cornerstone University, Ibadan, Oyo State, Nigeria

**Keywords:** COVID-19, nosocomial, Africa

## Abstract

The pandemic of Coronavirus disease 19 is not abating since the outbreak began in December 2019. Africa is currently experiencing a surge after an initial low incidence and nosocomial infections could be contributing to this. A dominant factor responsible for this is a weak healthcare system because of many years of neglect due to abysmal budgetary allocation to the sector. The testing capacity for COVID-19 diagnosis in Africa is grossly inadequate coupled with a severe shortage of personal protective equipment and inadequate infectious diseases expert. These factors exposed the frontline health workers and patients to the hazard of nosocomial infection with the attendants´ morbidity and mortality. Deliberate efforts need to be made toward reducing nosocomial COVID-19 infection.

## Introduction

The Coronavirus disease 19 (COVID-19) pandemic, caused by SARS-CoV-2 virus, shows no sign of abating since the outbreak was first reported in Wuhan City of China in December 2019. There are now about 13 million confirmed cases worldwide, which has resulted in 574,464 deaths as of July 15, 2020. On February 14, 2020, Africa recorded the first case in Egypt and currently has about 600,000 cases, with over 13,000 deaths [1]. The devastating effects of the COVID-19 outbreak have been enormous in both the developed and the developing world. Africa, comprising mostly developing countries, initially had a low incidence of COVID-19, but this has been increasing, at an alarming rate with the highest daily frequency reported recently. South Africa, Egypt, and Nigeria have the highest cumulative confirmed cases on the continent so far. The weak healthcare system in Africa presents a challenge of nosocomial infections. According to the World Health Organization (WHO) estimate before COVID-19 pandemic, the pooled prevalence of healthcare-associated infections (HAI) was greater in low and middle-income countries or developing countries (10%) than in high income or developed countries (7.6%) [2]. There have been many cases of COVID-19 among healthcare workers with attendant mortality. Nosocomial SARS-CoV-2 infection could also affect patients in the hospital for other ailments [3]. The unique nature of COVID-19 with an evolving understanding of the transmission, pathogenesis, and pathophysiology makes infection control a challenge. A review and meta-analysis reported from China had estimated the occurrence of nosocomial infection for SARS-CoV-2, SARS and MERS to be 44%, 36% and 56%, respectively [4].

## Commentary

In May 2020, there was a report of nosocomial SARS-CoV-2 infections affecting 80 members of staff and 39 patients in a hospital in South Africa [5]. Special attention must be paid to SARS-CoV-2 nosocomial spread in Africa in other to tame theCOVID-19 pandemic. One of the main challenges of nosocomial infection in Africa is the weak health system and infrastructure. This is due to many years of neglect by most governments as evidenced by the meager budgetary allocation to the sector and poor utilization of the allocated resources due to widespread corruption and mismanagement [6]. This directly impacts hospitals' capacity to address infection prevention and control (IPC) in their facilities. The implementation of IPC programs is sub-optimal or lacking in many health facilities, especially in sub-Saharan Africa. There is a dearth of competent personnel and infrastructure to carry out effective IPC in most hospitals in Africa. Therefore, the emergence of COVID-19 met an unprepared system. Notwithstanding, the Africa Center for Disease Control has recently been scaling up capacity building and implementing programs, especially with the pandemic's occurrence. However, there is still a long way to go. The ability to make a prompt diagnosis of COVID-19 is another issue that has a significant role in infection control. Delay in diagnosis could affect the appropriate management of patients vis-à-vis proper isolation to control infection. Real-time Polymerase Chain Reaction (RT-PCR) is the gold standard for the detection of SARS-CoV-2, the virus that is responsible for COVID-19. However, the prohibitive cost of the equipment and the need for at least Biosafety Level (BSL) 2 laboratories limit the possibility of large-scale testing, which is essential in infection prevention and control [7]. For example, at the beginning of the COVID-19 outbreak in Nigeria in February, there were only four laboratories that could test forSARS-CoV-2; there has been a recent expansion to 40 over four months under the coordination of Nigeria Centre for Disease Control (NCDC). However, the testing capacity of most of the laboratories is very limited, as many are makeshift laboratories and the number of laboratories still falls short of the number and capacity that is required for a country with a population of over 200 million. The inability to provide confirmatory results on time has the potential to fuel disease spread within health facilities, which could affect both patients and staff. It is a common occurrence to have patients receive positive results for COVID-19 after spending days on a general ward leading to disease transmission among other patients and medical staff. In some situations, it is not uncommon to see a suspected patient housed in a general ward due to a lack of special isolation or holding wards. This further stress the unprepared nature of African’s health system for emerging infectious diseases.

Furthermore, the inadequate provision of personal protective equipment (PPE) is another factor that could influence nosocomial COVID-19, especially among the health workforce that is in the frontline. Africa is one of the worst-hit with the global shortage of PPE following the pandemic. Most countries in Africa depend on importation, including donations from international organizations and philanthropists to meet their PPE need. This has led to the rationing of the limited number of available PPE with consequent inadequate protection of frontline health workers resulting in the occurrence of high rates of infection among healthcare personnel. The appropriate and rational use of limited PPE by healthcare workers is also another challenge as many are not familiar with the process of donning and doffing of PPE which could be the source of nosocomial infection. This is further compounded by the possibility of infection being transmitted in the community when infected hospital personnel carry the pathogen to their family members, neighbor, and guests. Unchecked morbidity and mortality among healthcare personnel will result in increasing lost man-hours and a reduction in the healthcare workforce. For political reasons, it may be difficult to obtain accurate data on COVID-19 infection among healthcare workers which further impair the ability to appropriately manage the situation. There have been reports of declaration of industrial action by healthcare worker unions to protest insufficient PPE in hospitals and other factors that would have motivated the healthcare workers in the line of duties such as life insurance and other incentives.

The inadequate number of infectious disease experts, including those that specialize in infection control in many parts of Africa, is a cause for concern as it relates to SARS-CoV-2 nosocomial transmission. Even though many infectious diseases including those with the potential to cause public health emergency of international concern (PHEIC) such as Lassa fever and Ebola viral disease with prior large-scale outbreaks, are prevalent in Africa. The investment and development of specialists in every aspect of infectious diseases have been grossly deficient. This includes experts in molecular biology and infectious disease immunology needed to conduct researches that address outstanding questions in infectious disease transmission and host response to infectious diseases including COVID-19 in the African setting. Hospital infection prevention and control experts, among others, are also desperately needed. Deliberate funding and investment within Africa will be required to address this [8]. There is an observation that COVID-19 prevalence may be relatively less reported in Africa in the early part of the pandemic, which may be related to under-diagnosed that is widespread in the continent than the rest of the world. The surge in the last few weeks could be an ominous sign that will require concerted efforts to. Improved testing coverage and generation of a more representative data will reveal the true burden of COVID-19 in Africa. Efforts are in progress to conduct seroprevalence surveys and support COVID-19 response in Nigeria and 3 other African countries by the Center for International Health, Education, and Biosecurity (CIHEB) in the Institute of Human Virology, Baltimore, Maryland in collaboration with African partner agencies and government ministries. Beyond this, there is a need to improve on the health infrastructure, significantly expand diagnostic facilities for COVID-19, and scale up the provision of personal protective equipment to healthcare personnel across the continent. In the long term, deliberate investment to improve on the existing system and build personnel capacity in infectious diseases should be planned. Prevention of nosocomial infection is imperative at protecting hospital staff and patients and reduce associated morbidity and mortality.
